# Pesticide-Induced Inflammation at a Glance

**DOI:** 10.3390/toxics11110896

**Published:** 2023-10-31

**Authors:** Monica Lopes-Ferreira, Luiz Rogério Ludwig Farinha, Yasmin Stefanie Oliveira Costa, Felipe Justiniano Pinto, Geonildo Rodrigo Disner, João Gabriel dos Santos da Rosa, Carla Lima

**Affiliations:** Immunoregulation Unit, Laboratory of Applied Toxinology (CeTICs/FAPESP), Butantan Institute, São Paulo 05503900, Brazil; luizrogeriofarinha@gmail.com (L.R.L.F.); yasmin.costa.esib@esib.butantan.gov.br (Y.S.O.C.); felipe.pinto.esib@esib.butantan.gov.br (F.J.P.); geonildo.disner.esib@esib.butantan.gov.br (G.R.D.); joao.rosa.esib@esib.butantan.gov.br (J.G.d.S.d.R.); carla.lima@butantan.gov.br (C.L.)

**Keywords:** herbicide, fungicide, insecticide, immune system, immunotoxicity, xenobiotics

## Abstract

The increasing number of studies reporting the risks of the exposure to pesticides aligned with the intensified use of such hazardous chemicals has emerged as a pressing contemporary issue, notably due to the potential effects to both the environment and human health. Pesticides, while broadly applied in modern agriculture for pest control and crop protection, have raised concerns due to their unintended effects on non-target organisms. The immune system exerts a key role in the protection against the exposome, which could result in cellular imbalances and tissue damage through the inflammatory response. Pesticides, which encompass a diverse array of chemicals, have been linked to inflammation in experimental models. Therefore, the aim of this review is to discuss the increasing concern over the risks of pesticide exposure focusing on the effects of various chemical classes on inflammation by covering, as broadly as possible, different experimental approaches as well as the multiple or co-exposure of pesticides. Overall, pesticides potentially induce inflammation in different experimental models, manifested through skin irritation, respiratory impairment, or systemic effects. The connection between pesticides and inflammation highlights the importance of proper handling and regulation of these substances and underscores the need for research into safer and sustainable practices to reduce our reliance on synthetic pesticides and fertilizers.

## 1. Introduction

The increasing number of studies published in recent decades reporting the risks of the exposure to pesticides aligned with the intensified use of such hazardous chemicals has emerged as a pressing contemporary issue, notably due to the potential effects to both the environment and human health. According to the Food and Agriculture Organization [1] in 2022, there has been a significant increase in the global export and import of pesticides between 1990 and 2020. Notably, there has been a notable emphasis on high import rates from both America and Europe. In 2020, Brazil and the USA emerged as the leading countries in pesticide usage worldwide.

Although pesticides have been extensively used in industrial farming for pest control and crop protection, serious uncertainties have been raised due to their unintended effects on non-target organisms and ecosystems [2]. Among these concerns, the study of inflammatory toxicity responses or inflammation outcomes resulting from pesticide exposure has gained significant attention [3,4,5]. This line of investigation holds paramount importance as it sheds light on the potential adverse impacts of pesticides on human and animal health.

The immune system exerts a key role in the protection of organisms against potential antigens, including biological agents, like bacteria, viruses, and chemical agents that could result in cellular imbalances and tissue damage via the activation of the inflammatory response [6]. Pesticides can directly affect immune organs by inducing oxidative damage or altering the signaling pathways modulating their functions, resulting in the inhibition of serine hydrolases or esterases, which are crucial components of an effective immune system [7].

Inflammation is a fundamental biological response aimed at defending the body against harmful stimuli, such as pathogens, toxins, and tissue injury [8]. However, when this response becomes dysregulated or chronic, it can lead to a range of ensuing health problems. Pesticides, which encompass a diverse array of chemical compounds, have been linked to triggering inflammatory responses in experimental models and even in human populations. This raises a crucial question: could exposure to pesticides contribute to the development or exacerbation of inflammatory-related diseases in humans?

The inflammatory response induced by pesticides in the most affected target systems, i.e., skin, airway system, and gastrointestinal system, is a multifaceted process that underscores the importance of these barriers as the body’s first defense response. When pesticides come into contact with the organism, they can trigger a cascade of events. The mucous and epithelial membranes within these systems, for example, act as vigilant sentinels, releasing alarmins and other signaling molecules to initiate a swift and coordinated immune response. This response, involving various immune cells and cytokines, aims to neutralize the threat and repair any tissue damage, highlighting the remarkable complexity and efficiency of the body’s defense mechanisms [9]. Additionally, injured epithelial cells on the skin’s surface recognize the xenobiotics and release alarmins, such as interleukin-25 (IL-25), interleukin-33 (IL-33), thymic stromal lymphopoietin (TSLP), and high-mobility group box 1 (HMGB1) [10,11,12].

The release of these alarmins from the epithelium leads to the downstream production of interleukin-1-beta (IL-1β) that drives the recruitment of neutrophils. Recruitment of neutrophils from the bloodstream to inflamed tissues requires a carefully regulated cascade of binding interactions between adhesion molecules on leukocytes and endothelial cells. Neutrophil trafficking is orchestrated by adhesion molecules, such as β2 integrins, chemokines, and cytokines. Additionally, these molecules serve as danger signals, alerting the immune system to the presence of a threat. Simultaneously, mucus and epithelial membranes lining the airways and gastrointestinal tract act in a similarly manner, releasing signaling molecules. Simultaneously, inflammatory cytokines like IL-1β and tumor necrosis factor-alpha (TNF-α) amplify the inflammatory response. Over time, the immune system works to repair any damage caused by the pesticides. Nevertheless, excessive neutrophil recruitment and their activation result in the degranulation and release of their vast arsenal of hydrolytic (myeloperoxidase, elastase, and metalloproteinases), oxidative (reactive oxygen species (ROS)), pore-forming molecules, and cytokine (IL-1β and interleukin-17-A (IL-17A)) mediators into the extracellular medium, worsening inflammation. Furthermore, upon activation, neutrophils are able to excrete chromatin embedded with components from their cytoplasmic granules to form neutrophil extracellular traps (NETs) covered with enzymes and cytokines, leading to host tissue injury [13].

The extensive use of pesticides in agriculture, urban pest control, and other applications has led to their ubiquitous presence in the environment [14,15]. Because of their mobility and their ability to accumulate in the environment, these chemicals can enter the food chain, affect water sources, and even become airborne. While the acute toxicity of certain pesticides is well documented, the subtler, long-term effects, such as inflammatory responses, have gained increasing attention due to their potential to contribute to chronic health conditions. Understanding the mechanisms by which pesticides induce or modulate inflammation is essential for evaluating their overall safety and for designing effective risk-mitigation strategies.

To address these concerns, scientists are employing experimental models to investigate how different types of pesticides influence systems toxicology through inflammatory responses at the cellular and molecular levels. Moreover, epidemiological studies are being conducted to explore potential associations between pesticide exposure and inflammatory-related diseases in human populations. By comprehensively examining the inflammatory toxicity outcomes of pesticide exposure in non-human experimental models, researchers strive to provide evidence-based insights that can inform regulatory decisions, shape agricultural practices, and ultimately safeguard One Health.

The primary purpose of our study is to explore the pesticides impact on inflammatory processes. Our focus relies on a comprehensive literature review spanning the last five years, which investigates the main classes of pesticides and their associated effects in experimental models. Due to the risk of pesticide exposure, it is extremely important to understand the complex relationship between these chemicals and inflammation.

## 2. Methodology

**Figure 1:**
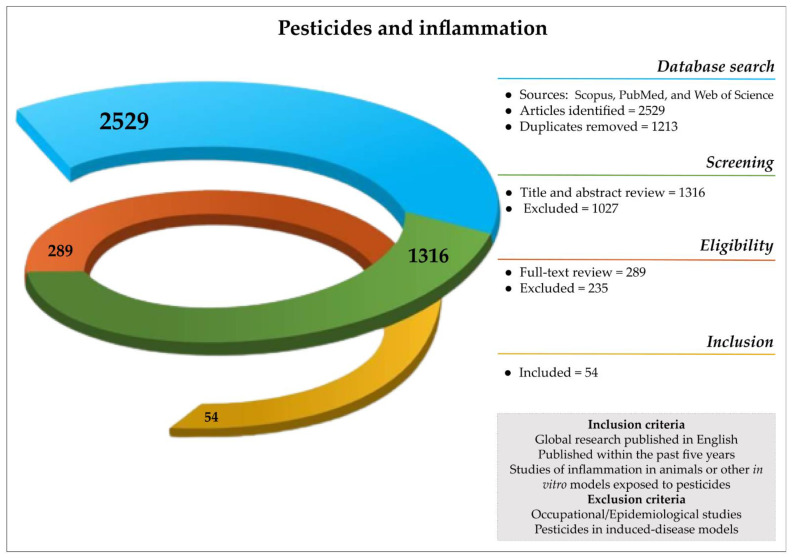
Spiral diagram of manuscript selection and inclusion–exclusion criteria applied for this review.

Based on the search term “(Pesticides OR agrochemicals) AND Inflammat*”, a search strategy was applied in the search platforms Scopus, PubMed, and Web of Science in April 2023. The selection underwent a series of eligibility steps (Figure 1) analyzed by double-blind review using the following as the inclusion criteria: articles published within 5 years, research published in English, and research articles reporting inflammation in experimental animal models or in vitro related to pesticide exposure regardless of the class. Articles were excluded when the pesticides were used to induce—or were combined with—a disease model to evaluate novel treatments and epidemiological/occupational studies due to the complexity to isolate phenomena. The initial search found 2529 publications in which, at the end, 54 papers fit the chosen requirements. We extracted data on the experimental model used, exposure level and route, inflammatory endpoints analyzed, main inflammatory outcomes observed, and the country of origin of the author responsible for the study (Supplementary Material, Table S1).

## 3. Results and Discussion

The current agricultural system aims to achieve increased production in a shorter time frame, and the development of large-scale agriculture has intensified pesticide use to enhance productivity and ensure economic efficiency [16]. Both industrialized and developing countries, such as China, the USA, Brazil, Argentina, France, and Canada, extensively employ pesticides to improve crop efficiency.

**Figure 2:**
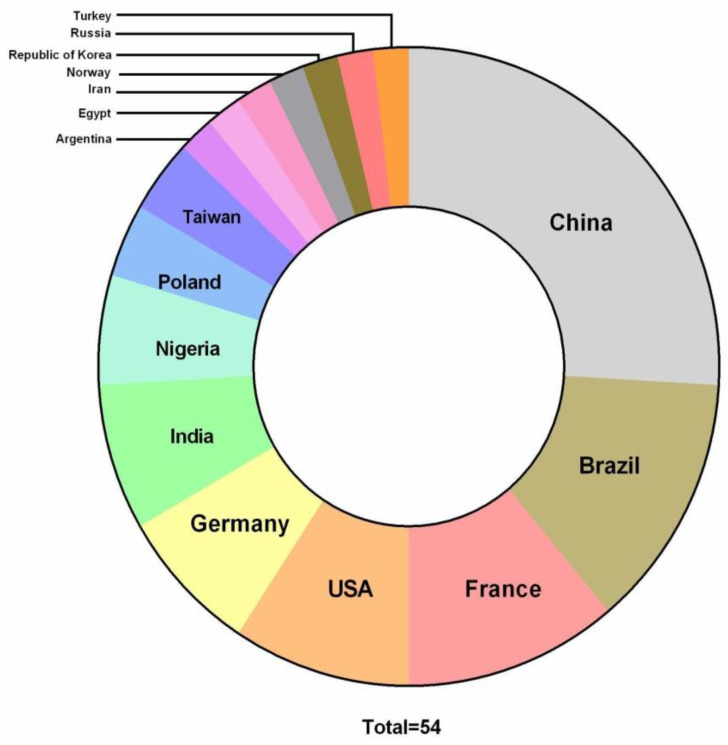
Home country of the studies about pesticides and inflammation in the last five years. The 54 articles included in the review were grouped according to the country where the last author is affiliated and where most of the research was carried out.

In this review, global studies relating pesticides to inflammation, performed in the last five years, were covered and compiled according to the last author affiliation. Naturally, the countries with major production and consumption of pesticides also have the most prolific scientific literature involving this matter (Figure 2).

Pesticide consumption reached 4.2 million metric tons in 2022, representing an approximate 80 percent increase compared to 1990 [1]. China was the largest pesticide-consuming country, using 1.76 million metric tons of pesticides, followed by the USA and Brazil, with 408 and 377 thousand metric tons consumed, respectively [1].

Most pesticides are non-selective toxicants, affecting both aquatic and terrestrial animals, as well as humans, as unintended targets [17]. Evidence suggests that certain pesticides accumulate in non-target organisms due to their persistent properties, and particular pesticide residues move through the food chain, affecting the entire ecosystem. Due to the continuous use of pesticides in agriculture, substantial quantities of these residues accumulate in the soil environment, posing significant risks of environmental contamination.

Concerning human exposure to pesticides, two different forms of exposure are recognized: occupational, where exposure occurs during the application process, and non-occupational, consisting of occasional exposure to pesticides through many ways.

These substances can penetrate tissues by breaching epithelial barriers, such as in the lungs, skin, and intestinal mucosa, leading to transient or permanent physiological alterations in directly affected tissues [18]. Direct skin contact is a common form of pesticide interaction, and absorption can vary depending on the pesticide formulation, influencing the level of tissue damage. Likewise, contact with the respiratory tract has deleterious effects on mucosal surfaces, and the same applies to the gastrointestinal tract, which is vulnerable to pesticide exposure from contaminated water, food, and other sources.

Pesticides are typically applied directly to crops, which can lead to their accumulation in plants, soil, and water bodies. Furthermore, when pesticides are released into crops, they can be absorbed into the soil through surface runoff and become a widespread and persistent source of pollution. Consequently, non-target populations can be exposed to pesticides and their residues. Most pesticides include in their formulation components that optimize the chemical performance. These substances, such as surfactants, provide more efficacy and high solubility; however, these molecules can present toxicity or synergistically interact with the active ingredient, increasing the pesticide toxicity.

**Figure 3:**
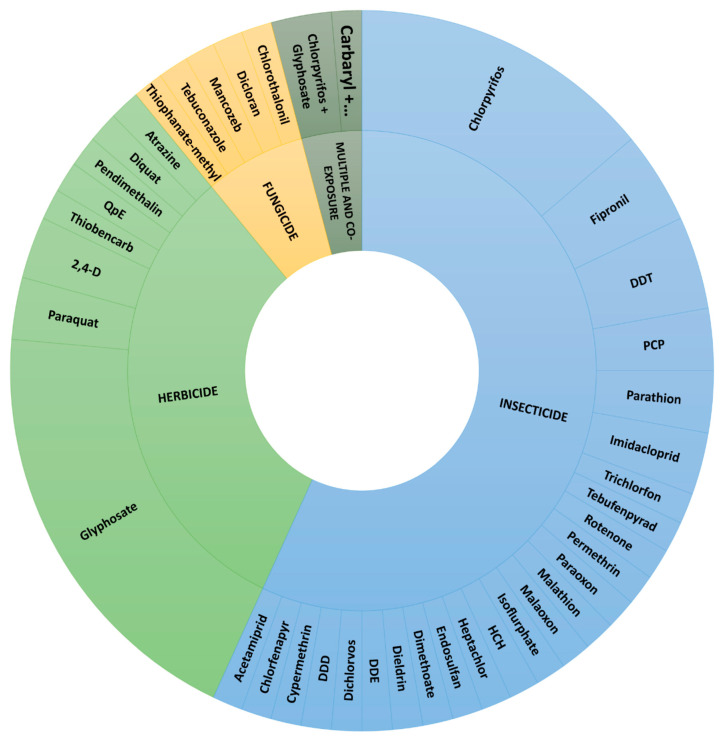
Distribution of tested pesticides. DDD: Dichlorodiphenyldichloroethane; DDE: dichlorodiphenyldichloroethylene; DDT: dichlorodiphenyltrichloroethane; HCH: hexachlorocyclohexane; PCP: pentachlorophenol; QpE: quizalofop-p-Ethyl; 2,4-D: 2,4-dichlorophenoxyacetic acid. Multiple and co-exposure: Chlorpyrifos + Glyphosate and Carbaryl + Dimethoate + Methomyl + Methyl parathion + Glyphosate + Triadimefon.

Our review covered several pesticides grouped by the class of experimental approach, as seen in Figure 3. Thus, the search resulted in 20 studies comprising eight different herbicides, twenty-six studies with 25 different insecticides, three studies focusing on five different fungicides exposure, and five studies with multiple or co-exposure to pesticides.

The potential bioaccumulation of pesticides in biological tissues, combined with the various possible effects of their components, offers multiple avenues for physiological damage caused by pesticides. Additionally, pesticides may induce toxicity through biochemical processes, such as oxidative stress and inflammation, leading to various adverse effects, including growth inhibition, increased mortality and teratogenicity, oxidative damage, apoptosis, and immune system disorders [19].

**Figure 4:**
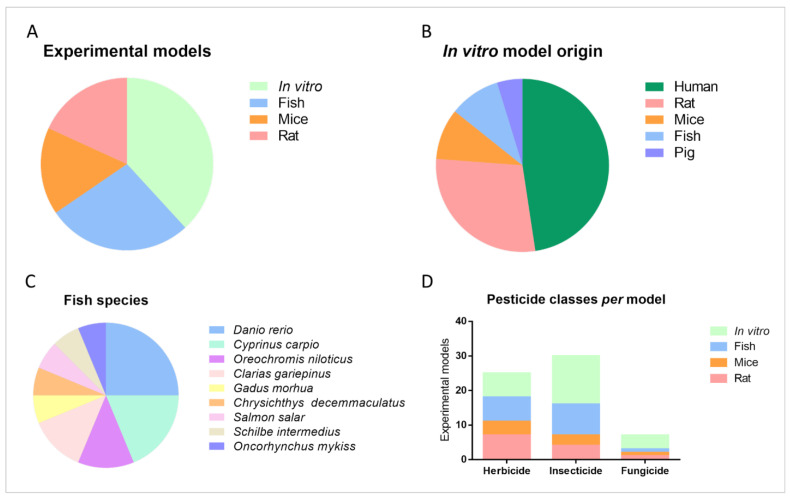
Breakdown of experimental models applied for the study of pesticide negative effects. (**A**) Experimental models most used; (**B**) discrimination of the origin of in vitro models; (**C**) discrimination of fish species assessed; (**D**) pesticide classes tested per model.

Experimental models play a pivotal role in understanding the effects of pesticides on inflammation. In our review, we found that four types of experimental models are primarily used: in vitro, the most tested experimental approach; fish; rats; and mice (Figure 4A). In vitro cell culture models involve growing cells in a controlled environment, allowing for the examination of direct effects of pesticides on cytokine production, oxidative stress, and cell viability. The research articles under this review used cell cultures from different organisms, the major source of cells came from humans (e.g., human keratinocytes or natural killer cells), followed by rodents, fish, and pigs (Figure 4B). Recently, fish have gained popularity as a model organism for toxicological studies, including those related to pesticides and inflammation. Fish are important specimens in the aquatic ecosystems and are considered excellent sentinels in biomonitoring since they are prone to direct exposure to chemical pollutants, especially those commonly verified in aquatic environments. Zebrafish, the most frequent fish used as an experimental model in this review (Figure 4C), present several advantages and allow for the real-time visualization of inflammatory responses. Three major classes of pesticides were studied by the selected articles, namely insecticides, herbicides, and fungicides. Figure 4D presents the proportion of applied experimental models against each pesticide class. In vitro models were predominantly used, followed by fish and murine models (Figure 4D).

### 3.1. Pesticide-Triggered Effects on the Immune System

Insecticides are designed to target the nervous systems of insects, disrupting their normal functions and ultimately leading to their demise. However, the potential impact of insecticides on non-target organisms, including humans, has raised concerns. Studies have shown that exposure to certain insecticides, such as organophosphates and pyrethroids, may trigger inflammatory responses in humans and animals. The study of the link between insecticides and inflammation often focuses on the measurement of inflammatory biomarkers. These include markers such as interleukins (e.g., IL-6, IL-1β) and TNF-α. Elevated levels of these biomarkers can indicate an inflammatory response following insecticide exposure.

Toxicants in sublethal concentrations have the potential to interfere with physiological processes, and pesticides like chlorpyrifos (CPF) can alter the expression of proinflammatory cytokines, as demonstrated by Zahran et al. (2018), who found that exposure to different sublethal concentrations of CPF upregulated proinflammatory cytokines IL-8 and TNF-α [20]. Additionally, Wu et al. (2021) reported increased levels of TNF-α in the brain tissue of zebrafish following fipronil exposure, a widely used broad-spectrum insecticide, indicating neuroinflammation [21]. Likewise, Souders II et al. (2021) documented increased levels of inducible nitric oxide (NO) synthase, cyclooxygenase-2, and TNF-α, supporting the hypothesis of pesticide-induced neuroinflammation [22].

Liang et al. (2019) documented a positive association between CPF and obesity and insulin resistance [23]. The exposure to this insecticide through dietary intake resulted in an elevated expression of proinflammatory mediators in the liver, adipose tissue, ileum, and colon. The release of these proinflammatory cytokines incites tissue inflammation, suggesting that chlorpyrifos may induce gut inflammation in mice, contributing to metabolic conditions, such as insulin resistance and obesity, as the inflammatory mediators appear to interfere with insulin-receptor binding.

Similarly, another study investigating different dietary patterns revealed that high-fat diets could exacerbate gut inflammation caused by CPF due to an increase in proinflammatory mediators, such as TNF-α, IL-6, monocyte chemoattractant protein-1 (MCP-1), IL-1β, and INF-γ. Furthermore, the insecticide CPF led to gut epithelial tissue damage, resulting in bacterial translocation and more intense exposure to lipopolysaccharide (LPS), which contributes to heightened inflammation [24].

Epithelial barriers are continuously exposed to various aggressors, and the gastrointestinal epithelial barrier’s homeostasis relies on the balance of immune response, its components, and a healthy microbiota [25]. There is evidence that pesticides, such as CPF, can impact the intestinal barrier, as Huang et al. (2019) described a significant increase in TNF-α messenger RNA (mRNA) expression in the colon, suggesting that CPF intensified colon inflammation [26]. Chronic illnesses are generally rooted in chronic inflammation, and pesticides can activate innate immune functions, resulting in increased cytokine secretion, leading to localized microinflammations. The sustained release of pro-inflammatory cytokines plays a pivotal role in tumorigenesis, as demonstrated by Shah et al. (2020) in an ovarian cell culture, where prolonged exposure to organochlorine pesticides induced the secretion of proinflammatory cytokines, leading to chronic inflammation and contributing to tumorigenesis in diseases, such as ovarian cancer [27].

Recently, Chang et al. (2020) showed that the organophosphorus trichlorfon reduced transcriptional levels of claudin-2, occludin, and zonula occludens-1 (ZO-1), three tight junction genes in the intestinal mucosa, and also decreased villus height in chronically exposed common carps [28]. Similarly, Sun et al. (2018) observed significant decreases in villus length in rats exposed to another organophosphate, phoxim, along with the upregulated mRNA expression of TNF-α and IL-6 [18].

Zhao et al. (2021) investigated the effects of neonicotinoid insecticides on intestinal epithelium and inflammatory biomarkers under sublethal concentration exposure and found an upregulation of inflammatory factors, such as TNF-α and IL-1β [29].

Herbicides are chemical compounds designed to eliminate or reduce the growth of weeds in a specific crop [30,31], this occurs through the binding of herbicides to the plant’s enzyme action sites, which slows down or inhibits biochemical reactions and causes an accumulation of toxic substances in the cells [32]. Among all pesticides used worldwide, herbicides represent 60% of them [33]. Herbicides are frequently used worldwide, especially during rainy seasons when there is an increase in unwanted plant species in the cultivation fields [34]. This becomes an increasing concern since it has been demonstrated that herbicides can accumulate in the soil and contaminate water sources [35,36].

Furthermore, our review has shown that the use of herbicides can cause potential human health damage, particularly related to inflammation. Herbicides may disrupt intercellular junctions, compromise the epithelium, and lead to the expression of inflammatory cytokines, alarmins, and chemokines, recruiting immune cells such as neutrophils and macrophages (Supplementary Material, Table S1).

Glyphosate, for example, is the most widely used herbicide. Its mechanism of action involves inhibiting the 5-enolpyruvylshikimate-3-phosphate (EPSP) synthase enzyme, leading to the accumulation of toxic substances in vacuoles and the inhibition of the production of the amino acids tryptophan, tyrosine, and phenylalanine [31]. However, studies conducted on animal models or cells have shown that glyphosate induces inflammatory processes, increasing the expression and concentration of inflammatory cytokines, such as interleukin-6 (IL-6), IL-1β, and TNF-α, followed by an increase in the number of immune cells, like neutrophils and macrophages [37,38,39].

Pesticides and their components act through various mechanisms. For instance, the herbicide glyphosate presents surfactants in its formulations, which is responsible for its toxicity. Due to surfactants like polyethoxylated tallow amine (POEA), glyphosate-based herbicides can penetrate plants and act as herbicides. Nevertheless, the same can occur in animal cell membranes, allowing for bioaccumulation in plants and animals [40].

Moreover, glyphosate administration has been shown to alter gene expression mediated by nuclear factor kappa B (NF-kB), leading to the production of the superfamily of cytochrome P450 (CYP) proteins, caspase-3, and caspase-9 [41,42]. Inflammatory effects were primarily observed in the liver and intestines of rats and mice during experiments, organs closely involved in absorption and metabolism [37,43,44].

It is also noteworthy the study conducted by Buchenauer et al. (2022), which not only highlighted the significant inflammatory effects caused by glyphosate in mice, but also indicated that exposure of pregnant females affects the offspring’s immune system, resulting in immunosuppression (reduced interferon gamma, IFN-γ, and expression) and alterations in the intestinal microbiome [39].

Inflammatory reactions were also observed to the herbicide 2,4-D, which has a mechanism of action based on auxin hormone mimicry, which is responsible for plant growth [31]. In the studies included in this review, the pesticide was able to alter the recruitment of innate immune response cells and damage the skin and mouth epithelium of murine models [45,46].

Other herbicides found in this review also showed inflammatory effects and the activation of the innate immune system. Experiments conducted with animal models and cells demonstrated an increased expression of inflammatory cytokines (interleukin-6 (IL-6), IL-1β, TNF-α, and interleukin-8 (IL-8)), the recruitment of neutrophils and macrophages, an increased production of ROS, and generalized inflammation in the liver and intestines in animal studies [47,48,49,50,51].

Furthermore, farmers lose between 10 and 23 percent of their crops worldwide to fungal infection each year, despite widespread use of antifungals [52]. Fungicides are a category of pesticides designed to either eradicate fungi and their spores or inhibit their growth. They are usually applied in managing fungal threats to plants, comprising conditions like rusts, mildew, and blights. Fungicides exert their effects through diverse mechanisms, with many targeting the structural integrity of fungal cell membranes or disrupting the energy production processes within fungal cells. Despite their role in protecting crops, there are notorious concerns raised on potential negative effects on inflammation. Research suggests that exposure to certain fungicides can disrupt the sensitive balance of the immune system, leading to increased inflammation [53]. These chemicals may interfere with the functioning of immune cells, such as macrophages, by altering their cytokine production. Furthermore, fungicides can accumulate in the environment, contaminating water sources and food, which can contribute to long-term, low-level exposure for humans and wildlife.

Among all kinds of pesticides, fungicides were the class with the fewest studies included in the present review. It may be due to the fact that just a few highly cultivated crops, such as citrus fruits, are more intensively affected by fungal contamination. Nevertheless, the papers analyzed herein pointed to important deleterious effects on inflammation/inflammatory markers and diseases.

Tebuconazole, for example, a widely used triazole fungicide used to prevent and control a variety of fungal diseases by inhibiting the biosynthesis of ergosterol in fungi, caused colonic inflammation in mice in a gut-microbiota-dependent manner [54]. This fungicide increased *Akkermansia* abundance, leading to metabolic profile disorder and consequently impaired the intestinal barrier. The disruption of gut barrier integrity impairs its function and induces inflammation. Due to inflammation, Meng et al. (2022) reported cell infiltration in colon tissue and increased expression levels of TNF-α, IL-6, ΙFΝ-γ, Toll-like receptor 4 (ΤLR4), IL-1β, MCP-1, and interleukin-22 (IL-22) [54].

Another study evaluated the effects of dicloran photodegradation products on human inflammatory skin disease using a 3D skin model [55]. Dicloran (2,6-Dichloro-4-nitroaniline) is a fungicide used on a variety of fruits and vegetables as well as on conifers and various ornamentals. Top uses included celery, head lettuce, and leaf lettuce. The dicloran phototoxicity assessment indicated that photodegraded dicloran and its intermediate products resulted in epidermal inflammation in vitro through the induced expression of pro-inflammatory-related genes. Notably, C-C motif chemokine ligand 4 (*ccl4*), C-X-C motif chemokine ligand 2 (*cxcl2*), *il6*, and matrix metallopeptidase 9 (*mmp9*) demonstrated significantly upregulated expression levels (>200% of control with *p* < 0.05). This approach is of great importance since the application of pesticides generates a large number of chemical wastes, which, under environmental stress, undergo chemical structure modifications to potentially create more hazardous chemicals that deserve further investigation.

The third, and last, study evaluating fungicides dive into the elucidation of the inflammatory response after exposure to mancozeb, chlorothalonil, and thiophanate-methyl in RAW 264.7 cells (Weis et al., 2019). This triad of fungicides, in a similar manner, activated the inflammatory process by increasing the macrophage proliferative and pro-inflammatory cytokines production in macrophages (IL-1β, IL-6, and TNF-α), whereas the anti-inflammatory cytokine interleukin-10 (IL-10) levels decreased. Additionally, mancozeb, chlorothalonil, and thiophanate-methyl increased the levels of caspase 1, 3, and 8. Those results demonstrated that the fungicides studied exert immunomodulatory activities, thereby exacerbating the inflammatory response.

An important highlight for Mancozeb is that this widely used fungicide has been restricted or banned in several developed countries due to concerns about its potential health and environmental risks. However, it is still used in some underdeveloped regions because it is relatively inexpensive and effective against a range of fungal diseases. Mancozeb is a manganese/zinc ethylene-bis-dithiocarbamate fungicide that has been used since 1948 as a broad-spectrum fungicide. Due to its efficacy in a wide range of agricultural and industrial applications, including its use on major agricultural crops, its uses and production continue to grow even though several studies have associated its exposure and its main metabolite, ethylenethiourea (ETU), with a variety of health effects, including cancer, endocrine disruption, and reproductive toxicity [56].

Overall, while efforts are made to regulate fungicides, there is an ongoing debate about the adequacy of these regulations and the need for continued improvements to minimize contamination, toxicity, and environmental impact while ensuring crop protection and food security.

### 3.2. Risk of Pesticide Mixtures Contamination

In the field of environmental toxicology, under the One Health umbrella, the investigation of complex mixtures takes on paramount importance. This subject is of great concern when it comes to the contamination caused by pesticides since modern agriculture is one of the main pillars of the global economy and still relies heavily on chemical inputs for pest and weed control. However, this reliance has led to an alarming scenario where water, soil, and air are increasingly contaminated with a complex cocktail of pesticides, each tailored to specific crop needs. This growing contamination poses significant risks to both target and non-target organisms, making it imperative to delve into the complexities of these mixtures to understand their synergistic or antagonistic effects and the potential amplification of toxicity.

The multifaceted nature of pesticide mixtures demands thorough investigation due to their potential to exert synergistic effects that surpass the individual toxicity of their components or toxicological interactions that can lead to unpredictable effects. When multiple pesticides coexist in the environment, their combined impact on organisms more often than not can be greater than the sum of their individual toxicities. This phenomenon, known as synergism, can lead to unforeseen health and ecological consequences. Furthermore, complex pesticide mixtures can pose risks to non-target organisms, disrupting ecosystems and threatening biodiversity. Investigating these mixtures allows us to decipher their intricate interactions and assess the risks they pose to human health and the environment.

Throughout the reviewed papers, a few focused on the study of pesticide mixture. Interestingly, all the papers tested the combination of different pesticide classes, which is in consonance with realistic scenarios, whereas a rule-all class of pesticides could potentially contaminate the environment. The authors commonly point to the fact that standard research on pesticide toxicity does not address the action of mixtures of different pesticides, but rather their individual action. Therefore, even the studies aiming to establish the regulatory limits of use to guarantee sufficient protection to consumers should be performed considering the combined exposure of several substances, in addition to verifying safe doses for individual chemical substances. In the European Union, there is the legal requirement to account for cumulative effects for the usage of pesticide-active substances, which is more reasonable and considers the precautionary principle.

Corroborating this intricacy, Bonifacio & Hued, (2019) tested single and joint effects of chlorpyrifos and glyphosate-based pesticides on the fish *Cnesterodon decemmaculatus* [57]. In summary, circulatory disorders and inflammatory processes were significantly increased in the groups exposed to the pesticide mixture. Mixture treatments caused antagonism and potentiation in fish responses.

In one of the studies evaluating pesticide mixture, Docea et al. (2019) exposed rats during 12 months to low doses of a cocktail of 13 substances present in pesticides and food additives [58]. Increasing inflammation was found, notably in male rats, resulting from the exposure to medium to high doses. There was a significant, dose-dependent decrease in immune cells in groups of male and female rats exposed to medium and high doses of the mixture. Females treated with low doses of the mixture showed an increase in the concentration of TNF-α and IFN-γ increased in male rats from medium dose groups.

These studies reinforce the need to update the mono-chemical risk assessment approach to determine safe limits, considering real exposure scenarios. Investigating complex mixtures is not merely a scientific pursuit, it is a crucial step toward mitigating the harmful impacts of pesticide contamination on human health. By understanding how these mixtures interact and affect organisms, we can develop more informed policies and strategies to safeguard our environment and secure a healthier environment. A secondary difficulty is that the number and composition of potential pesticide mixtures is often unknown and changes over time, and the precise toxicity mechanism of many pesticides, is still unknown [59].

## 4. Conclusions

**Figure 5:**
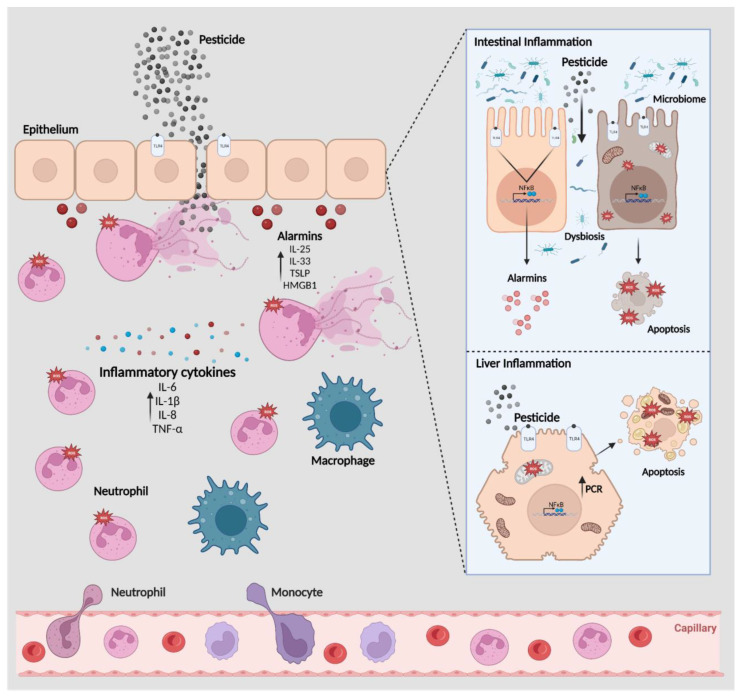
Representation of typical pesticide-induced innate inflammatory response. Created with BioRender.

Inflammation is a complex biological response that plays a crucial role in the body’s defense against harmful stimuli, including pesticides (Figure 5). The exposure to pesticides disrupts cell homeostasis, leading to cell injury and may also work as an antigen, activating Toll-like receptors in epithelial cells’ surface. This aggression incites the release of alarmins, and chemotactic and immune-activating proteins, such as IL-33, IL-25, TSLP, and HMGB1. Alarmins carry out neutrophil recruitment to the site, along with macrophages, and the consequent degranulation of these cells release proinflammatory cytokines, such as IL-6, IL-8, IL-1β, and TNF-α. Also, the neutrophil activity results in the production of high levels of ROS, implicating in more intense cell aggression and death. In the intestinal epithelium, the pesticide-derived injuries lead to dysbiosis and microbiome translocation, intensifying the local inflammatory response. Moreover, pesticide-induced TLR-activated cells, such as intestinal cells and hepatocytes, elevate ROS production, which contribute to cell degeneration and inflammation. While inflammation is typically a protective response, chronic, or excessive inflammation has been associated with a higher risk of inflammatory diseases, including autoimmune disorders like rheumatoid arthritis and inflammatory bowel disease.

Humans can potentially be exposed to pesticides residues, raising concerns about the impact on their health. Understanding the mechanisms by which pesticides can trigger inflammation and conducting rigorous risk assessments are essential steps toward minimizing their adverse effects on human health and the environment. Over reliance on pesticides can lead to issues such as the development of resistant strains and environmental harm. Integrated pest management (IPM) approaches, which combine chemical and non-chemical methods, are increasingly advocated to minimize chemical use while maintaining crop health. Sustainable agriculture practices and ongoing research into disease-resistant crop varieties are also important in addressing the challenges posed by the contamination of “pests” in agriculture.

It is essential to continue researching and monitoring the effects of pesticides on inflammation to ensure that their use in agriculture does not compromise human health or environmental integrity. Sustainable and responsible pesticide management practices are crucial in mitigating these potential negative consequences. Striking this balance is crucial to ensure the sustainability of agriculture while safeguarding human well-being.

## Supplementary Materials

The following supporting information can be downloaded at: https://www.mdpi.com/article/10.3390/toxics11110896/s1, Table S1: Summary of the papers included in the review of pesticides and inflammation highlighting the main effects on inflammatory endpoints.

## Abbreviations

2,4-D	2,4-dichlorophenoxyacetic acid
ccl4	C-C motif chemokine ligand 4
CPF	chlorpyrifos
cxcl2	C-X-C motif chemokine ligand 2
CYP	cytochrome P450
DDD	dichlorodiphenyldichloroethane
DDE	dichlorodiphenyldichloroethylene
DDT	dichlorodiphenyltrichloroethane
EPSP	5-enolpyruvylshikimate-3-phosphate
ETU	ethylenethiourea
HCH	hexachlorocyclohexane
HMGB1	high-mobility group box 1
IFN-γ	interferon gamma
IL-10	interleukin-10
IL-17A	interleukin-17-A
IL-1β	interleukin-1-beta
IL-22	interleukin-22
IL-25	interleukin-25
IL-33	interleukin-33
IL-6	interleukin-6
IL-8	interleukin-8
IPM	integrated pest management
LPS	lipopolysaccharide
MCP-1	monocyte chemoattractant protein-1
mmp9	matrix metallopeptidase 9
mRNA	messenger RNA
NETs	neutrophil extracellular traps
NF-kB	nuclear factor kappa B
NO	nitric oxide
PCP	Pentachlorophenol
POEA	polyethoxylated tallow amine
QpE	Quizalofop-p-Ethyl
ROS	reactive oxygen species
ΤLR4	Toll-like receptor 4
TNF-α	tumor necrosis factor-alpha
TSLP	thymic stromal lymphopoietin
UN	United Nations
USA	United States of America
ZO-1	zonula occludens-1
