# Supplementary material for: Pesticide-Induced Inflammation at a Glance
Source: Toxics. 2023 Oct 31;11(11):896. doi: 10.3390/toxics11110896 (PMC10675742; doi:10.3390/toxics11110896)
Supplement: Supplementary file 1 [file toxics-11-00896-s001.zip › toxics-2674908-supplementary.pdf]

Table S1: Summary of the papers included in the review of pesticides and inflammation highlighting the main effects on inflammatory endpoints.

| Class | Name | Inflammatory effect | Reference | Country | Experimental model |
|---|---|---|---|---|---|
| HERBICIDE | Glyphosate | Increased levels of pro-inflammatory cytokines IL-6 and TNF-α in the liver of Glyphosate exposed rats. The perinatal exposure increased the phosphorylation of p65NFκB | Rieg *et al*. (2022) | Brazil | *Rattus norvegicus* |
| | | The level of IL-1β, C3 and F13 in the liver increased following GBH exposure. | Qi *et al.* (2023) | China | *Mus musculus* |
| | | Direct exposure of dams induced a slight eosinophilic infiltration in the BAL and a significantly increased TH2 cytokine production. Th2 cytokines IL-13, IL-5 and Th17 cytokine IL-17 and Th1 cytokine IFN-γ were reduced in the female F1 indicating a more general immunosuppressive function. Furthermore, an altered gut microbiota was observed. | Buchenauer *et al.* (2022) | Germany | *Mus musculus* |
| | | Signs of intestinal inflammation such as celiac disease, increase in IELs. | Panza *et al.* (2021) | Brazil | *Mus musculus* |
| | | Increases of CYP1C1, CYP1B1, and CPY2C, cytokine secretion (IL-1β, IL-8, IL-10, IFN-γ, | Wang et al. | China | Spleen lymphocytes |

| | | | | | |
|---|---|---|---|---|---|
| | | TNF-α), inflammatory factors (NF-κB, cox-2), and the expression of miR-203 and the PI3K/AKT pathway | (2020) | | (*Cyprinus carpio*) |
| | | Up-regulation of pro-inflammatory cytokines IL-1β, IL-6, TNF-α and mRNA expression of iNOS and COX-2. | Bai *et al.* (2022) | China | *Sus scrofa domesticus* intestinal epithelial cells (IPEC-J2) |
| | | Reduction of the red blood cells and increased white blood cells (immature neutrophils) count was observed indicating the presence of inflammation. | Bojarski *et al.* (2022) | Poland | *Cyprinus carpio* |
| | | C-reactive protein in liver, cytokines IL-1β, TNF-α, IL-6, and inflammatory response marker, and prostaglandin–endoperoxide synthase were upregulated in liver and adipose of rats exposed to higher doses. | Pandey; Dabhade; Kumarasamy (2019) | India | *Rat* |
| | | mRNA expression levels of IL-1β, IL-6, TNF-α, MAPK3, NF-κB, and Caspase-3 were increased after glyphosate exposure. | Tang *et al.* (2020) | China | *Sprague Dawley* rats |
| | | Research revealed that GLY exposure also promoted expression of NF-κB, iNOS, IL-1β, IL-6, IL-8, and TNF-α; altered the levels of IL-10 and TGF-β, indicating that GLY | Ma *et al*. (2019) | China | *Cyprinus carpio L.* |

| | | | | | |
|---|---|---|---|---|---|
| | | exposure induced inflammatory response in the fish gills. Additionally, we found that GLY exposure activated apaf-1 and bax and inhibited bcl-2, induced caspase-9 and caspase-3 expression and caused remarkable histological damage in the fish gills. | | | |
| | | Pure glyphosate did not induce higher mortality but reduced interleukin-1*β*. | Du-Carrée et al., (2022) | France | *Oncorhynchus mykiss* |
| | 2,4-Dichlorophenoxyacetic acid | Chronic oral and inhalation exposure increase the presence and intensity of polymorphonuclear and/or mononuclear inflammatory infiltrate and individual cell necrosis. | Parizi *et al.* (2020) | Brazil | *Rattus norvegicus* |
| | | Even after acute exposure, the herbicide 2,4-D had the potential to damage the oral epithelium. Two animals in the SG and one in the MCG group presented a moderate inflammatory process with the presence of mononuclear cells. The other animals presented a mild inflammatory mononuclear infiltrate. | Parizi *et al.* (2020) | Brazil | *Mus musculus* |

| | | | | | |
|---|---|---|---|---|---|
| | Paraquat | Elevated levels of TNF-$\alpha$, IL-6 and IL-1β in exposed adult rats Zn/PQ or PQ/Zn as compared with controls. | Mittra *et al* (2020) | India | *Rattus norvegicus* |
| | Quizalofop-P-ethyl (QpE) | Inflammation-related cytokine genes of *NF-kB, IL-1β, IL-8, IFN* and *cc-chem* were affected after QpE exposure. Significant increase of *NF-κB* can be activated by TNF-$\alpha$. | Zhu *et al.* (2022) | China | *Danio rerio* |
| | Thiobencarb | After thiobencarb exposure inflammatory cells infiltration were observed in the interstitium of the ovaries. | Elias *et al.* (2020) | Iran | *Clarias gariepinus* |
| | Atrazine | Decrease in the expression of miR-181a-5p in lymphocytes exposed to Atrazine target TNF- $\alpha$ (1.4 – 1.6) while regulating HK2. NF-κB inflammatory pathway and the levels of glycol metabolism related genes were upregulated. | Cui *et al.* (2019) | China | Spleen lymphocytes (*Cyprinus carpio*) |
| | Pendimethalin | Change in the chymotrypsin level was observed. Pendimethalin induces oxidative damage and partially reduces the pancreatic enzyme levels and causes toxic effects when the pancreas becomes inflamed. | Arici *et al.* (2020) | Turkey | Human pancreatic cells (PANC-1) |

| | | | | | |
|---|---|---|---|---|---|
| | | Significantly induced oxidative damage, while levels of IL-6 and IL-8 did not change. | | | |
| | Diquat | Induced inflammatory response involves p53 signaling via activation of NF-kB role by various cytokines, chemokines, adhesion molecules, acute phase proteins, inducible effector enzymes, and regulators of cell proliferation and apoptosis. | Choi *et al.* (2018) | Republic of Korea | *Rattus norvegicus* PC12 cells |
| | Paraquat and Glyphosate | Micronucleated cells were significantly higher in the erythrocytes of *O. niloticus* exposed to the higher concentration of paraquat at day 14, both subacute concentrations of paraquat at day 28, and lower concentration of glyphosate at days 14 and 28 compared to the other treatments and controls. | Aribisala *et al*. (2022) | Nigeria | *Oreochromis niloticus* |
| | | Chlorpyrifos induced modifications of tight junctions and mucin gene expressions, leading to an increased epithelial permeability or inflammatory state. Whichever treatment received by mothers, plasma cytokines levels (IL-1β, IL-6 and TNF-α) measured at young adult age were | Guibourdenche *et al*. (2021) | France | *Rattus norvegicus* |

| | | | | | |
|---|---|---|---|---|---|
| INSECTICIDE | Chlorpyrifos | not different. | | | |
| | | Expression of inflammatory mediators (TNF-α, PAI-1, IL-1β, and MCP-1) in the ileum and colon confirmed that chlorpyrifos treatment could induce gut inflammation. | Liang *et al.* (2019) | China | *Mus musculus* |
| | | Increased MPO activity was accompanied by elevation of MDA (product of lipid peroxidation) indicating induction of inflammation and oxidative stress. | Adedara *et al.* (2018) | Nigeria | *Rattus norvegicus* |
| | | Increased expression of TLR4 mRNA, resulted in production of inflammatory cytokines, such TNF-α,IL-6, MCP-1, IL-1β, and IFN−γ. | Chen *et al.* (2023) | China | *Mus musculus* |
| | | Increased expression levels of IL-6, TNFα, and iNOS. The mRNA expression of NLRP3 inflammasome genes ASC, Caspase1, and NLRP3 were dramatically promoted by CPF, and the mRNA expression levels of mature IL-1β and IL-1 were also significantly increased by CPF. | Liu *et al.* (2023) | China | *Cyprinus carpio* |
| | | Matrix metalloproteinases increased by pro-inflammatory cytokines. (pro-hydroxy-pro) | Olsvik *et al.* (2019) | Norway | *Salmon salar* and |

| | | | | | |
|---|---|---|---|---|---|
| | | inflammation mediated induction of collagen breakdown indicates a possible inflammation. | | | *Gadus morhua* |
| | | Levels of TNF-α, IL-6, and IL-8 were upregulated in both head kidney and spleen in tilapia exposure to Chlorpyrifos. | Zahran *et al.* (2018) | Germany | *Oreochromis niloticus* |
| | | T-cell population in the blood and spleen declined in all CPF-exposed groups without affecting the proportion of $CD4^+$ and $CD8^+$ cells. Dietary CPF exposure disturbed CD4+ T cell polarization by suppressing Treg cells, which failed to control neutrophil-mediated inflammation. CPF-exposed mice had higher colonic TNF-α mRNA expressions, plasma haptoglobin levels, and luminal IgG concentration. | Huang *et al.* (2019) | Taiwan | *Mus musculus* T-cell, C57BL/6 |
| | | Alteration in the expression levels of apoptotic gene caspase-3 and inflammatory genes (TNF-α and IL-1 β). Increased rate of DNA damage as well as histopathological lesions in liver tissues. The mRNA levels of caspase-3, IL-1B, and TNF-a were super-expressed. | Refai *et al*. (2021) | Egypt | *Rattus norvegicus* |

| | | | | | |
|---|---|---|---|---|---|
| | Fipronil | Inflammation in the brain tissue of adult zebrafish. Decreased SOD2 and increase of TNF-α, caspase-3, after exposure. Quantified cerebral TNF-α/β-Tubulin ratio was higher in the zebrafish fipronil-exposed group. | Wu *et al.* (2021) | Taiwan | *Danio rerio* |
| | | Increased levels of inducible NO synthase, cyclooxygenase-2, and TNF-α which pointed to an inflammatory response. Caspase 3/7 activity was significantly increased after 6 and 12 h after fipronil exposure. | Souders II *et al.* (2021) | United States | Rat primary immortalized mesencephalic dopaminergic cells (N27) |
| | Dichlorvos | High levels of $H_2O_2$ and TNF were observed in the DDVP group increasing the inflammatory response. Increase of the expression of MHC class II molecules and strong tumoricidal activity. | Camargo et al. (2018) | Brazil | *Mus musculus* |
| | Isoflurphate | Induced inflammatory cytokines (IL-1β, TNF-α, and IL-8) and brain wide activation of microglia cells towards an M1-like inflammatory phenotype. Increases IL-8 and IL-1β transcripts. IL-4 cytokine was increased in the brains of larvae exposed. | Somkhit *et al*. (2022) | France | *Danio rerio* |

| | | | | | |
|---|---|---|---|---|---|
| | Imidacloprid | Increasing transcriptional levels of various pro-inflammatory factors, *e.g.*, IL-1β, IL-6, IL-8, TNF-α. | Luo *et al*. (2021) | China | *Danio rerio* |
| | | IMI exposure significantly increased intestinal permeability of male Wistar rats exposed to an oral dose, followed by elevated serum levels of endotoxin and inflammatory biomarkers (TNF-α, IL-1β) without any variation in body weight. Furthermore, a disturbance of the PXR-NF-kB-p65-MLCK signaling pathway was observed. | Zhao *et al.* (2021) | China | Wistar rats and Human intestinal epithelial cell line (Caco-2) |
| | Trichlorfon | Downregulation and upregulation of NTPDase and ADA activities, which contribute to the immune and pro-inflammatory deleterious cycle and its interaction with the P2X7 purine receptor contributes to the inflammatory mediators. | Baldissera *et al*. (2018) | Brazil | *Schilbe intermedius* |
| | Pentachlorophenol | Increase of IL-1β, IL-8, IL-6, MCP-1/CCL2, CCL5 and TNF-α by immune cells. Including NK-κB, STAT3, and p38 MAPK. An increase in transcription of TLR4 suggests the possibility that HMGB1 and/or | Thota *et al.* (2022) | United States | Humans cell lines HepG2 and A549 |

| | | | | | |
|---|---|---|---|---|---|
| | | Hsp70 induce inflammatory response by engaging TLR4. DAMPs in PCP induced inflammatory response | | | |
| | Chlorfenapyr (C), Acetamiprid (A) and the mixture (AC) | TNF-$\alpha$ responded substantially to pesticide exposure in all treated groups and was significantly increased in the following order: chlorfenapyr > acetamiprid. The results showed that pesticide exposure activated IL-6 and IL-1β expression to varying degrees. IL-6 in the groups treated with acetamiprid alone and combined drugs was markedly increased but not significantly affected by chlorfenapyr treatment alone. In contrast to IL-6, the IL-1β content increased sharply upon different degrees of exposure and was significantly promoted especially in the combination group. | Liu *et al.* (2021) | China | *Mus musculus* |
| | Cypermethrin and Dimethoate | Brain damages were exhibited as spongiosis, infiltration of inflammatory cells, focal area of liquefactive necrosis and distortion of brain architecture. | Okogwu *et al.* (2022) | Nigeria | *Clarias gariepinus* |
| | | Liver-like exposure to pesticides showed an | | | |

| | | | | | |
|---|---|---|---|---|---|
| | Dichlor-odiphenytrichloroethane (DDT) and Permethrin (PMT) and their mixture | increase in the production of lipids and carbohydrates and a decrease in amino acids and Krebs cycle intermediates, reflecting in hepatic inflammation. Aumento do Cxcl10 (pro inflammatory cytokine). Response to inflammation processes (Nfkb, IFN, TGF-β, p53, GSH) and liver regeneration (TGF-β) were also extracted consistently with IPA analysis. | Jellali *et al*. (2021) | France | Rat liver organ-on-chip |
| | Dichloro Diphenyl Dichloroethylene (DDT), Endosulfan, Heptachlor | Increased IL-1β, IL-6, and TNF-α, in HOSE cells after pesticides exposure induces epithelial ovarian cancer. | Shah *et al.* (2022) | India | Ovarian epithelial cells |
| | Rotenone, Tebufenpyrad | Increased expression of iNOS. Pesticide induced glial inflammation with direct correlation ENS (enteric nervous system) inflammation to CNS inflammatory manifestations. EGCs exposed to 1 μM Rot or Tebu for 6 h showed increased mRNA expression of the proinflammatory proteins NOS-2, IL-6 and TNFSF12 (TNF Superfamily Member 12). | Palanisamy *et al.* (2022) | United States | *Rattus norvegicus* (enteric glial cells) |

| | | | | | |
|---|---|---|---|---|---|
| | Malathion, Parathion, Paraoxon, Malaoxon | Exposure to parathion and paraoxon caused an increase of the cytokines IL-6, GM-CSF, VEGF and MIP-1 $\alpha$; increased expression of these cytokines indicates pulmonary inflammation. A decrease in resazurin was observed for paraoxon, malathion, and malaoxon. | Tigges *et al.* (2022) | Germany | *Rattus norvegicus* precision-cut lung slices (PCLS) |
| | Hexaclorociclohexano (HCH), Diclorodifenildicloroetileno (DDE), Dieldrin | E-cadherin and inflammatory mediators such as TNF-$\alpha$, IL-1β, and IL-6 were overexpressed after exposure. | Shah *et al.* (2020) | India | Epithelial cells of the human ovary |
| | Pentachlorophenol (PCP), Dichlorodiphenyltrichloroethane (DDT) | These compounds were able to stimulate IL-6 production from cell preparations containing T lymphocytes plus monocytes within 6 h of exposure. DDT exposure increased levels of mRNA for IL-6 indicating effects on either increased transcription or decreased breakdown of IL-6 mRNA. | Martin *et al.* (2019) | United States | Human immune cells (NK cells) |
| | Dichlorodiphenyltrichloroethane | After 24 hours of culture exposure, only glycine was significantly decreased and a trend decrease of cysteine, histidine and | Jellali *et al.* (2018) | France | *Rattus norvegicus* (Cell culture and |

| | | | | | |
|---|---|---|---|---|---|
| | (DDT), Permethrin (PMT) | methionine were observed indicating oxidative stress. Increase of fatty acid and lipids levels after DDT treatment | | | Biochips microfluid) |
| FUNGICIDE | Tebuconazole | Exposure promotes colitis, increases Akkermansia, destroys the intestinal barrier function and induces colonic inflammation. The expression levels increased TNF-$\alpha$, IL−6, IFN−$\gamma$, TLR4, IL-1$\beta$, MCP-1, IL-22. | Meng *et al.* (2022) | China | *Mus musculus* |
| | Dicloran | Human keratinocyte differentiation amplified the activation of dermal fibroblast cells, which were significantly impacted after four-hours irradiation dicloran. 1,4-benzoquinone is one of dominant intermediate products after four-hour degradation resulted in the epidermal inflammation in human skin. | Xu *et al.* (2018) | United States | Human skin keratinocytes |
| | Mancozeb (MZ), Chlorothalonil (CT), Thiophanate-methyl (TM) | MZ, CT, and TM exposure activated the inflammatory process by an increased macrophage proliferative and cytokine production (IL-1$\beta$, IL-6, TNF-$\alpha$, and IFN-$\gamma$). MZ, CT, and TM pesticides increased the levels of caspase 1, 3, and 8. | Weis *et al.* (2019) | Brazil | Mice macrophage cells (RAW 264.7) |
| | Chlorpyrifos, | High dose of Chlorpyrifos, Dithianon, and | | | Human monocyte- |

| | | | | | |
|---|---|---|---|---|---|
| MULTIPLE EXPOSURE AND CO-EXPOSURE | Thiacloprid (Insecticide)<br><br>Dithianon, Captan, Thiophanate, Boscalid (Fungicide) | Captan inhibited ROS production and pro-inflammatory cytokines TNF-α and IL-1β. Captan and Dithianon increased mRNA levels of antioxidant enzymes, NQO1 and HMOX1. The studied pesticides were unable to trigger the production of IL-1β, TNF-α pro-inflammatory cytokines nor the production of the IL-10 anti-inflammatory cytokine compared to control cells whatever doses. | Parny *et al.* (2022) | France | derived macrophage cells |
| | Chlorpyrifos (Insecticide)<br><br>Glyphosate (Herbicide) | Pesticides increased TNF-α and IFNγ production, which would suggest directing the immune response towards more inflammation. | Mendler *et al.* (2020) | Germany | Mucosal-associated invariant T cells |
| | Glyphosate (GLY) (Herbicide)<br><br>Deltamethrin (DEL) (Insecticide)<br><br>Chlorothalonil (CHL) (Fungicide) | The largest disintegration of the cell membrane was due to the action of 100 µg/mL DEL for U-937 and CHL at 1 µg/mL for HL-60. 3,600 µg/mLof GLY caused significant peroxidation of U-937 cells' lipids. CHL-induced inflammation in both types of cells tested. DEL and GLY also induced antioxidant activity in cells. | Barbasz *et al.* (2020) | Poland | Human immune cells (U-937 and HL-60) |

| | | | | | |
|---|---|---|---|---|---|
| | Carbaryl, Dimethoate, Methomyl, Methyl parathion (Insecticide)<br><br>Glyphosate (Herbicide)<br><br>Triadimefon (Fungicide) | TNF-α protein expression was up-regulated in male treated with medium and high doses. Medium dose treatment induced an up-regulation of IFN-γ protein expression in males as compared to the reference group. | Docea *et al*. (2019) | Russia | *Rattus norvegicus* |
| | Chlorpyrifos (Insecticide) + Glyphosate (Herbicide) | Circulatory disturbances ($HI_{Liv.Rp1}$) increased after mixture of pesticides while inflammatory processes ($HI_{Liv-Rp3}$) increased after only Chlorpyrifos and the mixture (Glyphosate + Clorfox) | Bonifácio & Hued (2019) | Argentina | *Chrysichthys decemmaculatus* |

**List of abbreviations:**

2,4-D – 2,4-Dichlorophenoxyacetic acid
ADA – Adenosine deaminase
AKT – Protein kinase B
ASC – Apoptosis-associated Speck-like protein containing a caspase-1 recruitment domain
BAL – Bronchoalveolar lavage
cc-chem – CC chemokine
CCL5 – CC motif chemokine ligand 5

CCR9 – CC motif chemokine receptor 9
CD103 – Dendritic cell 103
CD4+ – CD4 T lymphocytes
CD8+ – CD8 T lymphocytes
CHL – Chlorothalonil
CNS – Central nervous system
COX-2 – Cyclooxygenase-2
CPF – Chlorpyrifos
CT – Chlorothalonil
CXCL10 – CXC motif chemokine ligand 10
CYP1B1 – Cytochrome P450 family 1 subfamily B member 1
CYP1C1 – Cytochrome P450, family 1, subfamily C, polypeptide 1
CPY2C – Cytochrome P450, family 2, subfamily C
DAMP – Damage-associated molecular pattern
DDE – Diclorodifenildicloroetileno
DDT – Dichlorodiphenyltrichloroethane
DDVP – Dichlorvos
DEL – Deltamethrin
DMSO – Dimetilsulfóxido
EGCs – Enteric glial cells
ENS – Enteric nervous system
GBH – Glyphosate-based herbicide
GLY – Glyphosate
GM-CSF – Granulocyte-macrophage colony-stimulating factor
GSH – Glutathione S-transferase
H2O2 – Hydrogen peroxide
HCB – Hexachlorobenzene

HCH – Hexaclorociclohexano
HK2 – Hexokinase 2
HMGB1 – High-Mobility Group Box 1
HMOX1 – Heme oxygenase 1
HOSE cells – Human ovarian surface epithelial cells
Hsp70 – Heat shock protein 70
IEL – Intraepithelial lymphocyte
IFN−γ – Interferon gamma
IgG – Immunoglobulin G
IL-1β – Interleukin 1 beta
IL-6 – Interleukin 6
IL-8 – interleukin 8
IL-22 – Interleukin-22
iNOS – Inducible nitric oxide synthase
IPA – Ingenuity pathway analysis
MCG – middle 2,4-D concentration group
MCP-1 – Monocyte chemoattractant protein-1
MCP-1/CCL2 – Monocyte chemoattractant protein-1
MDA – 3,4-methylenedioxyamphetamine
MHC – Major histocompatibility complex
MIP-1 α – Macrophage inflammatory protein-1 alpha
MPO – Myeloperoxidase
MZ – Mancozeb
NK-κB – Nuclear factor kappa beta
NLRP3 – NOD-like receptor family pyrin domain containing 3
NO – Nitric oxide
NOS-2 – Nitric oxide synthase 2

NQO1 – NADPH dehydrogenase quinone 1
NTPDase – Nucleoside triphosphate diphosphohydrolase
P2X7 – Purinergic P2X7 receptor
p38 MAPK – p38 mitogen-activated protein kinase
p53 – Tumor protein p53
PAI-1 – Plasminogen activator inhibitor-1
PCP – Pentachlorophenol
PMT – Permethrin
PQ – paraquat
QpE – Quizalofop-P-ethyl
ROS – Reactive oxygen species
Rot – Rotenone
SG – saline group
SOD – Superoxide dismutase
STAT3 – Signal transducer and activator of transcription 3
Tebu – Tebufenpyrad
TGF-β – Transforming growth factor beta
Th2 – T helper 2 cells
TLR4 – Toll-like receptor 4
TM –Thiophanate-methyl
TNF-$\alpha$ – Tumor necrosis factor -$\alpha$
TNFSF12 – TNF Superfamily Member 12
Treg cells – Regulatory T cells
VEGF – Vascular endothelial growth factor
Zn – zinc
